# P-2126. ‘SPECIFIC’ First-In-Human Clinical Trial of ^68^Ga-triacetylfusarinine (TAFC) Siderophore PET/CT for Detection of Invasive Aspergillosis

**DOI:** 10.1093/ofid/ofae631.2282

**Published:** 2025-01-29

**Authors:** Beatrice Z Sim, Sidney Levy, Mohammad Haskali, Clemens Decristoforo, Hubertus Haas, Brittany Emmerson, Usama Kamil, Monica Slavin, Michael Hofman, Abby Douglas

**Affiliations:** Peter MacCallum Cancer Centre, National Centre for Infections in Cancer, University of Melbourne, Melbourne, Victoria, Australia; Peter MacCallum Cancer Centre, Royal Melbourne Hospital, University of Melbourne, Melbourne, Victoria, Australia; Peter MacCallum Cancer Centre, University of Melbourne, Melbourne, Victoria, Australia; Medical University of Innsbruck, Innsbruck, Austria, Innsbruck, Tirol, Austria; Medical University of Innsbruck, Innsbruck, Austria, Innsbruck, Tirol, Austria; Peter MacCallum Cancer Centre, Melbourne, Victoria, Australia; Peter MacCallum Cancer Centre, Melbourne, Victoria, Australia; National Centre for Infections in Cancer, Peter MacCallum Cancer Centre, Melbourne, Victoria, Australia; Peter MacCallum Cancer Centre, University of Melbourne, Melbourne, Victoria, Australia; National Centre for Infections in Cancer, Peter MacCallum Cancer Centre, Melbourne, Victoria, Australia

## Abstract

**Background:**

Invasive *Aspergillus* spp. infections (IAI) confer high morbidity and mortality in immunocompromised patients. Diagnosis often requires invasive and risky sampling. ^18^F-fluorodeoxyglucose positron emission tomography with computed tomography (FDG-PET/CT) is useful in diagnosis but is non-specific.

Siderophores are pathogen-specific iron chelators which are a promising radiolabelling target for IAI diagnosis. *Aspergillus fumigatus* secretes 2 siderophores, fusarinine C and triacetylfusarinine (TAFC), which are upregulated during IAI. Gallium-68 (^68^Ga)-labelled TAFC is attractive due to its short half life, rapid renal clearance, and low radiation risk. Preclinical mouse studies demonstrate diagnostic potential of ^68^Ga-TAFC PET/CT with specific visualisation of *A. fumigatus* infection.

**Figure 1:**
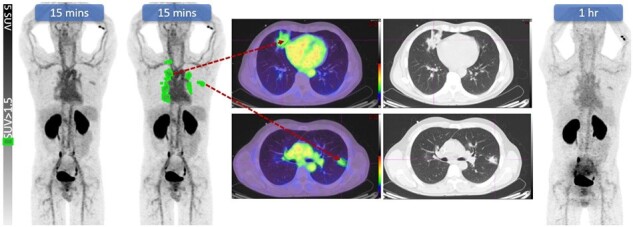
68Ga-TAFC-PET/CT in a patient with leukemia and probable pulmonary A. fumigatus IAI. Specific infective uptake of known pulmonary lesions at around blood pool level intensity (SUVmax 3.5, SUVmean 2.3). There was minimal liver and marrow uptake, with progressive washout to 60 minutes.

**Methods:**

We report the first result from a 10 patient pilot trial of ^68^Ga-TAFC PET/CT for IAI diagnosis (NCT06105411). Inclusion criteria are adults with pulmonary IAI within 2 weeks of diagnosis. Exclusion criteria are pregnant/lactating patients or those within a week of iron infusion.

^68^Ga-TAFC PET/CT was performed at 15, 62, and 209 minutes after IV 139 MBq ^68^Ga-TAFC administration. Findings pertinent to IAI and physiologic uptake were analysed.

**Results:**

The patient had probable *A. fumigatus* IAI in the setting of leukemia. Galactomannan and *Aspergillus fumigatus* PCR were positive on bronchoalveolar lavage fluid. Empiric antifungal treatment was commenced with fever resolution at 72 hours.

^68^Ga-TAFC-PET/CT was performed 10 days after treatment start and 4 days after microbiological diagnosis. Blood pool activity (SUVmax at 15/60/209 mins: 4.1/2.7/1.9) was demonstrated with minimal liver, spleen and gastrointestinal tract uptake (SUVmax 2.7), early renal excretion, and no marrow or other organ uptake. There was focal uptake in multiple pulmonary lesions (SUVmax 3.5), similar to blood pool activity, with synchronous washout over time. No adverse effects occurred.

**Conclusion:**

Very low background physiologic biodistribution was seen in the first participant in this study. Focal low grade pulmonary uptake was visualised, likely demonstrating specific activity for *A.fumigatus*. Trial recruitment continues.

**Disclosures:**

Abby Douglas, MBBS, Gilead Sciences: Honoraria

